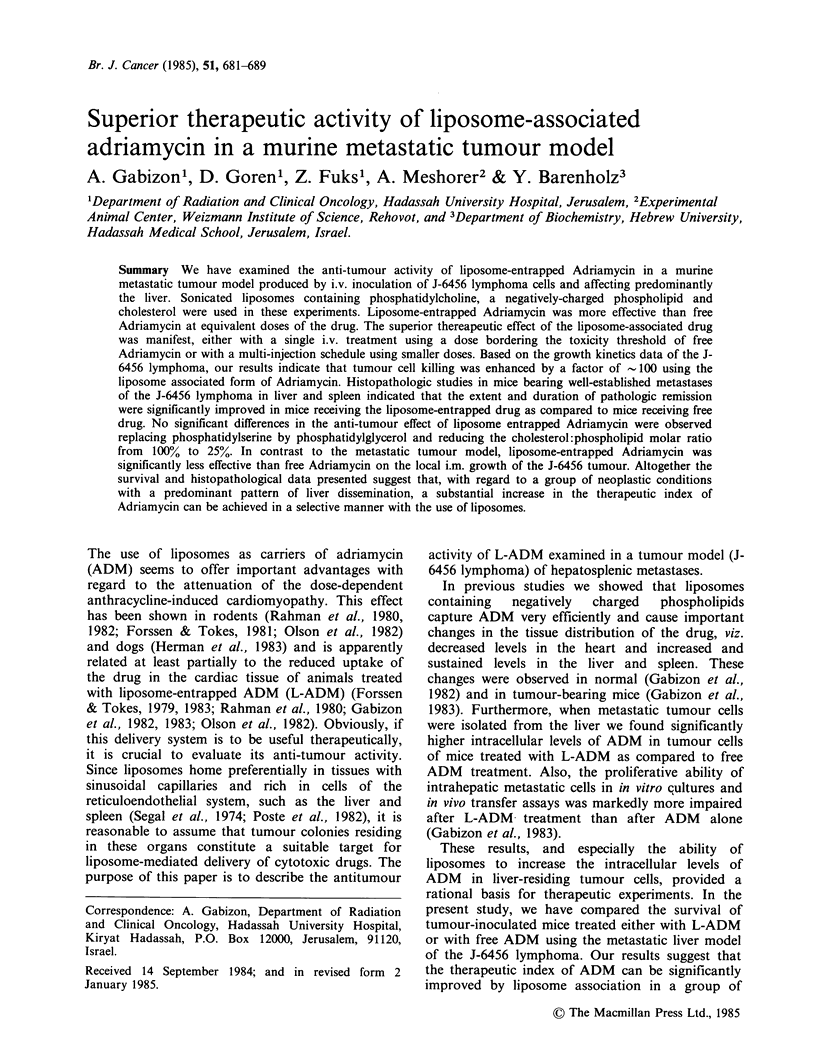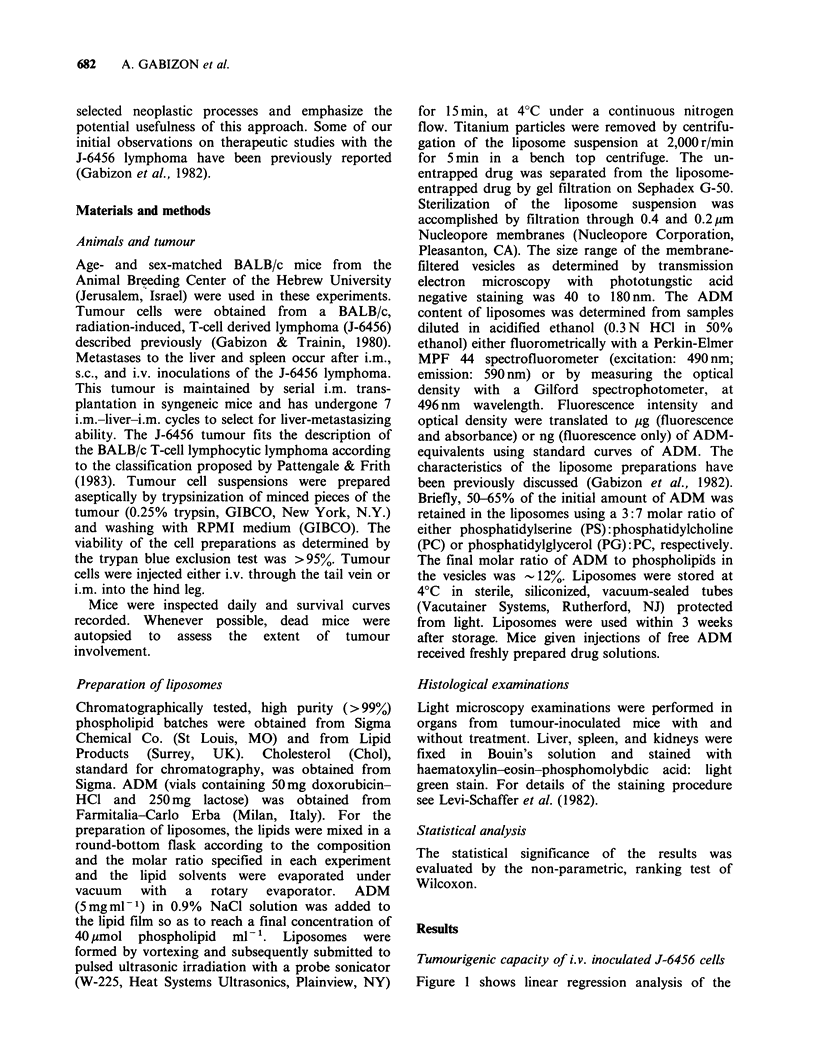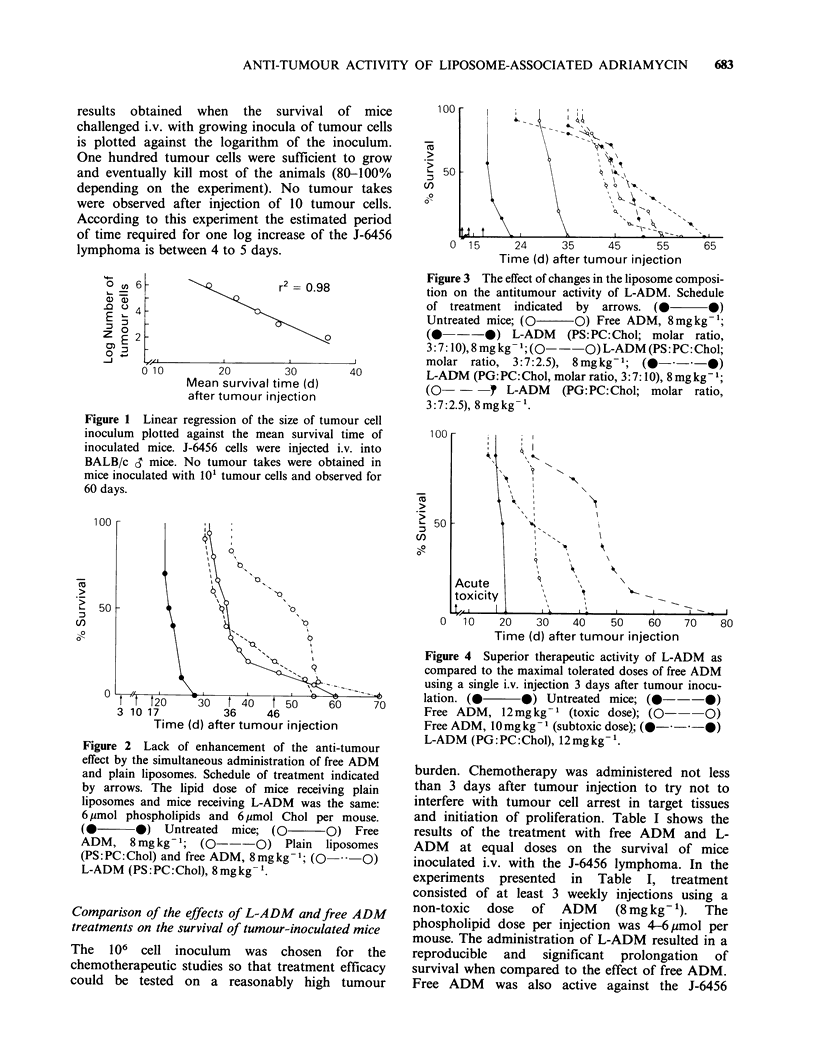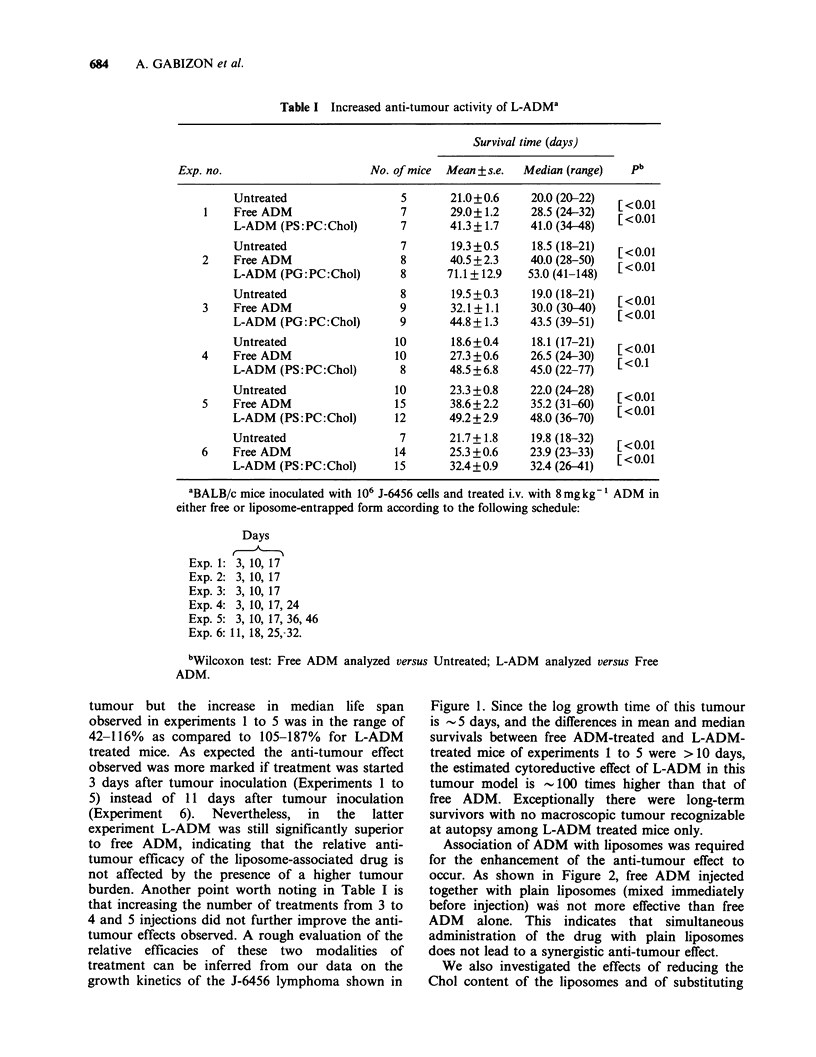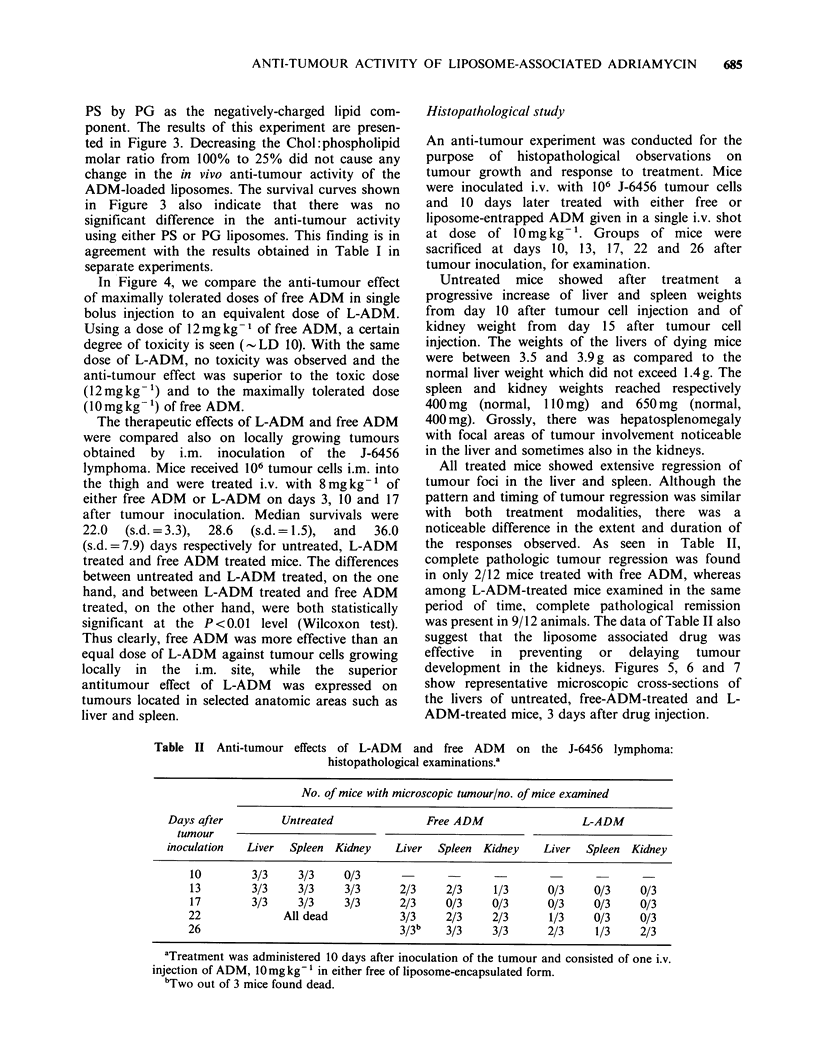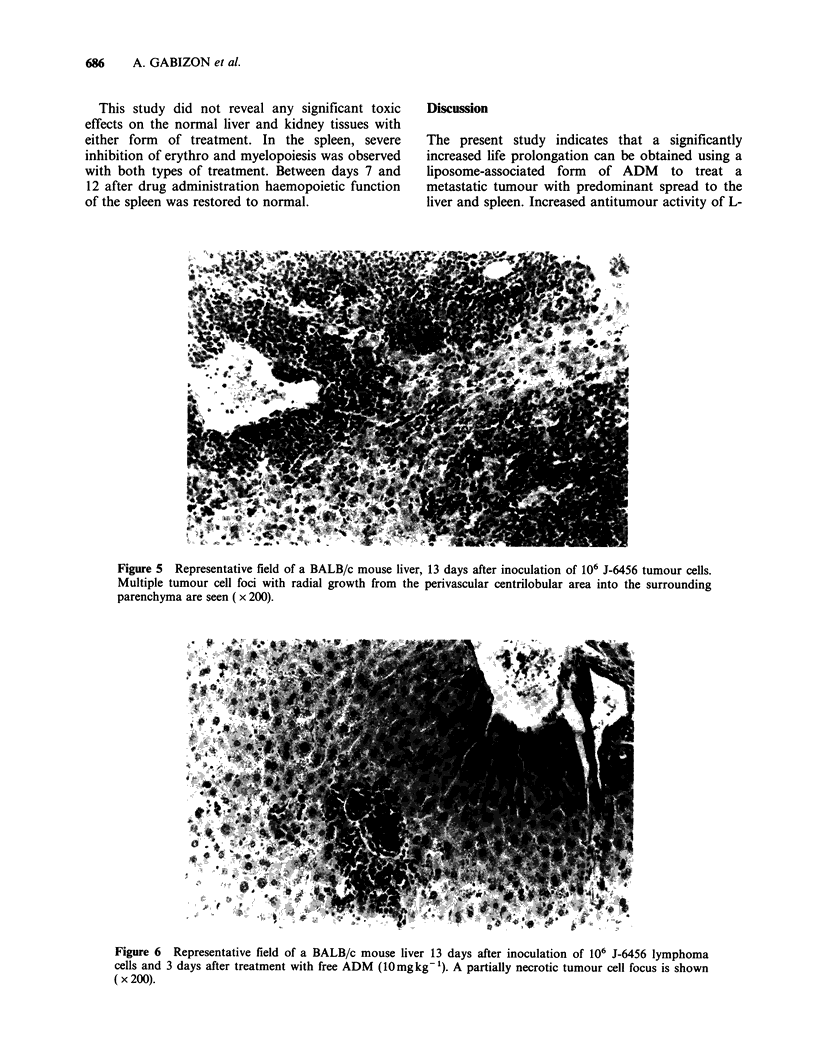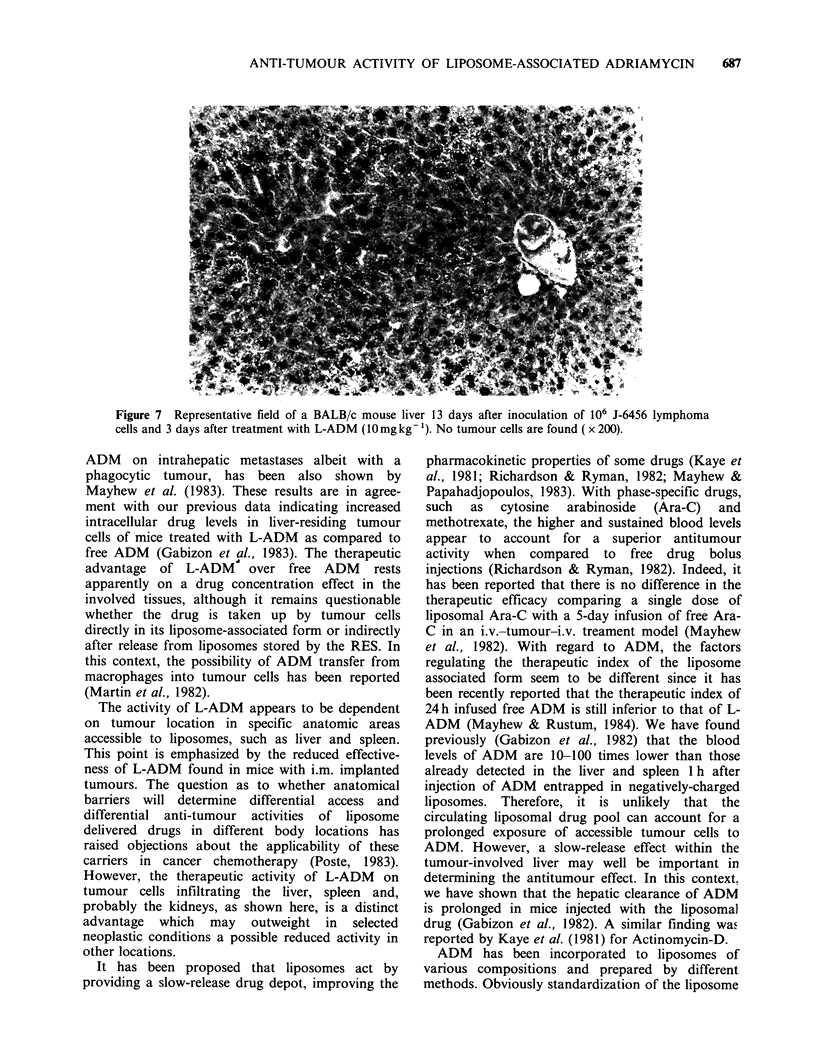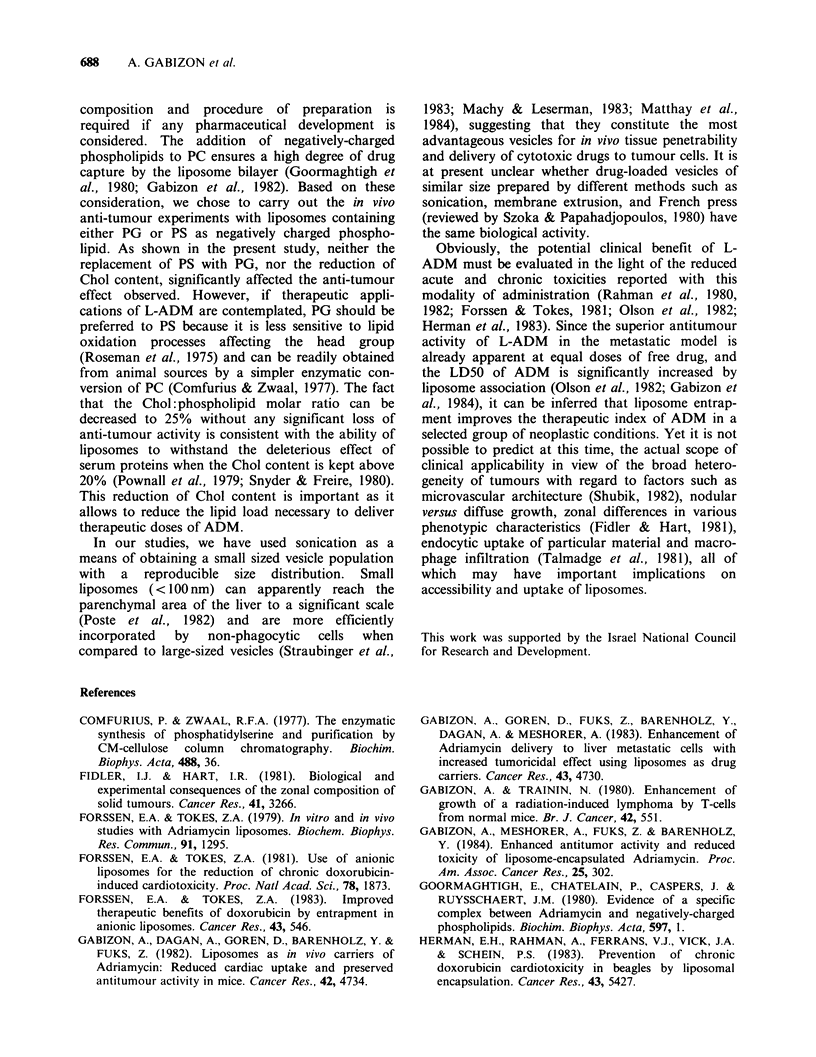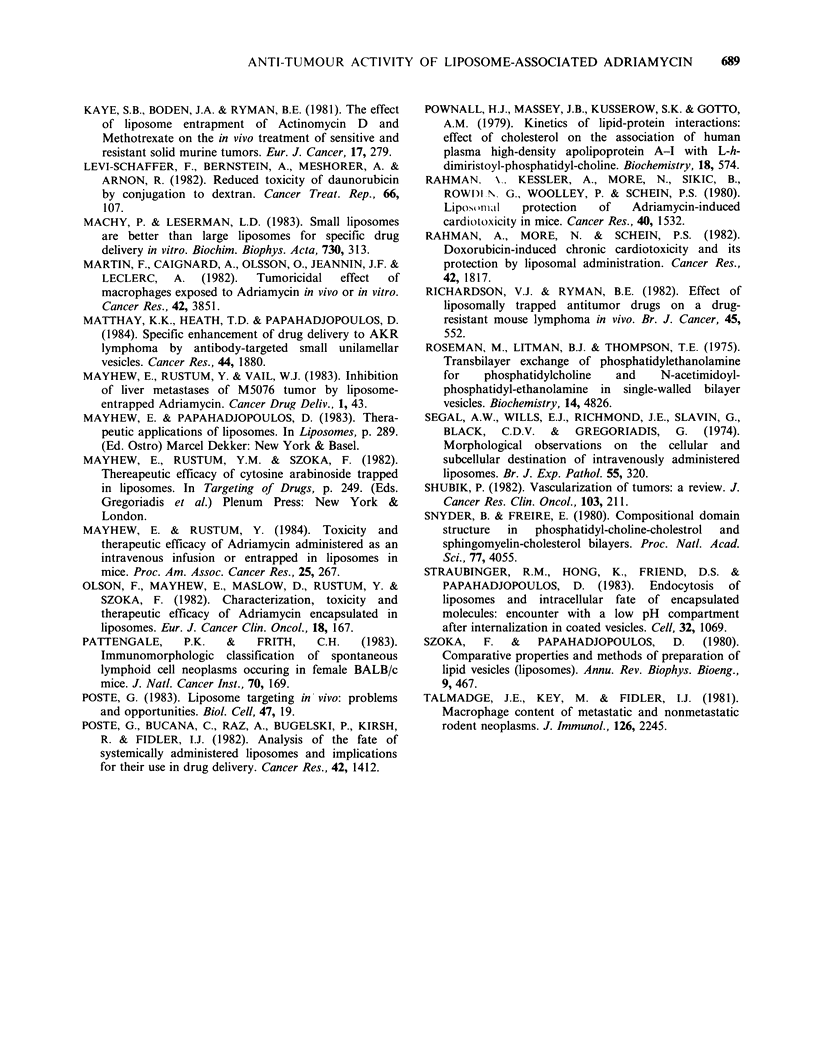# Superior therapeutic activity of liposome-associated adriamycin in a murine metastatic tumour model.

**DOI:** 10.1038/bjc.1985.103

**Published:** 1985-05

**Authors:** A. Gabizon, D. Goren, Z. Fuks, A. Meshorer, Y. Barenholz

## Abstract

**Images:**


*Br. J. Cancer* (1985), **51**, 681–689

### Superior therapeutic activity of liposome-associated adriamycin in a murine metastatic tumour model

A. Gabizon[1], D. Goren[1], Z. Fuks[1], A. Meshorer[2] & Y. Barenholz[3]

[1]*Department of Radiation and Clinical Oncology, Hadassah University Hospital, Jerusalem,* [2]*Experimental Animal Center, Weizmann Institute of Science, Rehovot, and* [3]*Department of Biochemistry, Hebrew University, Hadassah Medical School, Jerusalem, Israel.*

**Summary** We have examined the anti-tumour activity of liposome-entrapped Adriamycin in a murine metastatic tumour model produced by i.v. inoculation of J-6456 lymphoma cells and affecting predominantly the liver. Sonicated liposomes containing phosphatidylcholine, a negatively-charged phospholipid and cholesterol were used in these experiments. Liposome-entrapped Adriamycin was more effective than free Adriamycin at equivalent doses of the drug. The superior thereapeutic effect of the liposome-associated drug was manifest, either with a single i.v. treatment using a dose bordering the toxicity threshold of free Adriamycin or with a multi-injection schedule using smaller doses. Based on the growth kinetics data of the J-6456 lymphoma, our results indicate that tumour cell killing was enhanced by a factor of ~100 using the liposome associated form of Adriamycin. Histopathologic studies in mice bearing well-established metastases of the J-6456 lymphoma in liver and spleen indicated that the extent and duration of pathologic remission were significantly improved in mice receiving the liposome-entrapped drug as compared to mice receiving free drug. No significant differences in the anti-tumour effect of liposome entrapped Adriamycin were observed replacing phosphatidylserine by phosphatidylglycerol and reducing the cholesterol:phospholipid molar ratio from 100% to 25%. In contrast to the metastatic tumour model, liposome-entrapped Adriamycin was significantly less effective than free Adriamycin on the local i.m. growth of the J-6456 tumour. Altogether the survival and histopathological data presented suggest that, with regard to a group of neoplastic conditions with a predominant pattern of liver dissemination, a substantial increase in the therapeutic index of Adriamycin can be achieved in a selective manner with the use of liposomes.

The use of liposomes as carriers of adriamycin (ADM) seems to offer important advantages with regard to the attenuation of the dose-dependent anthracycline-induced cardiomyopathy. This effect has been shown in rodents (Rahman *et al.*, 1980, 1982; Forssen & Tokes, 1981; Olson *et al.*, 1982) and dogs (Herman *et al.*, 1983) and is apparently related at least partially to the reduced uptake of the drug in the cardiac tissue of animals treated with liposome-entrapped ADM (L-ADM) (Forssen & Tokes, 1979, 1983; Rahman *et al.*, 1980; Gabizon *et al.*, 1982, 1983; Olson *et al.*, 1982). Obviously, if this delivery system is to be useful therapeutically, it is crucial to evaluate its anti-tumour activity. Since liposomes home preferentially in tissues with sinusoidal capillaries and rich in cells of the reticuloendothelial system, such as the liver and spleen (Segal *et al.*, 1974; Poste *et al.*, 1982), it is reasonable to assume that tumour colonies residing in these organs constitute a suitable target for liposome-mediated delivery of cytotoxic drugs. The purpose of this paper is to describe the antitumour activity of L-ADM examined in a tumour model (J-6456 lymphoma) of hepatosplenic metastases.

In previous studies we showed that liposomes containing negatively charged phospholipids capture ADM very efficiently and cause important changes in the tissue distribution of the drug, *viz.* decreased levels in the heart and increased and sustained levels in the liver and spleen. These changes were observed in normal (Gabizon *et al.*, 1982) and in tumour-bearing mice (Gabizon *et al.*, 1983). Furthermore, when metastatic tumour cells were isolated from the liver we found significantly higher intracellular levels of ADM in tumour cells of mice treated with L-ADM as compared to free ADM treatment. Also, the proliferative ability of intrahepatic metastatic cells in *in vitro* cultures and *in vivo* transfer assays was markedly more impaired after L-ADM treatment than after ADM alone (Gabizon *et al.*, 1983).

These results, and especially the ability of liposomes to increase the intracellular levels of ADM in liver-residing tumour cells, provided a rational basis for therapeutic experiments. In the present study, we have compared the survival of tumour-inoculated mice treated either with L-ADM or with free ADM using the metastatic liver model of the J-6456 lymphoma. Our results suggest that the therapeutic index of ADM can be significantly improved by liposome association in a group of

Correspondence: A. Gabizon, Department of Radiation and Clinical Oncology, Hadassah University Hospital, Kiryat Hadassah, P.O. Box 12000, Jerusalem, 91120, Israel.

Received 14 September 1984; and in revised form 2 January 1985.

© The Macmillan Press Ltd., 1985

selected neoplastic processes and emphasize the potential usefulness of this approach. Some of our initial observations on therapeutic studies with the J-6456 lymphoma have been previously reported (Gabizon *et al.*, 1982).

### Materials and methods

### *Animals and tumour*

Age- and sex-matched BALB/c mice from the Animal Breeding Center of the Hebrew University (Jerusalem, Israel) were used in these experiments. Tumour cells were obtained from a BALB/c, radiation-induced, T-cell derived lymphoma (J-6456) described previously (Gabizon & Trainin, 1980). Metastases to the liver and spleen occur after i.m., s.c., and i.v. inoculations of the J-6456 lymphoma. This tumour is maintained by serial i.m. transplantation in syngeneic mice and has undergone 7 i.m.–liver–i.m. cycles to select for liver-metastasizing ability. The J-6456 tumour fits the description of the BALB/c T-cell lymphocytic lymphoma according to the classification proposed by Pattengale & Frith (1983). Tumour cell suspensions were prepared aseptically by trypsinization of minced pieces of the tumour (0.25% trypsin, GIBCO, New York, N.Y.) and washing with RPMI medium (GIBCO). The viability of the cell preparations as determined by the trypan blue exclusion test was >95%. Tumour cells were injected either i.v. through the tail vein or i.m. into the hind leg.

Mice were inspected daily and survival curves recorded. Whenever possible, dead mice were autopsied to assess the extent of tumour involvement.

### *Preparation of liposomes*

Chromatographically tested, high purity (>99%) phospholipid batches were obtained from Sigma Chemical Co. (St Louis, MO) and from Lipid Products (Surrey, UK). Cholesterol (Chol), standard for chromatography, was obtained from Sigma. ADM (vials containing 50 mg doxorubicin-HCl and 250 mg lactose) was obtained from Farmitalia–Carlo Erba (Milan, Italy). For the preparation of liposomes, the lipids were mixed in a round-bottom flask according to the composition and the molar ratio specified in each experiment and the lipid solvents were evaporated under vacuum with a rotary evaporator. ADM ($5\,mg\,ml^{-1}$) in 0.9% NaCl solution was added to the lipid film so as to reach a final concentration of $40\,\mu mol$ phospholipid $ml^{-1}$. Liposomes were formed by vortexing and subsequently submitted to pulsed ultrasonic irradiation with a probe sonicator (W-225, Heat Systems Ultrasonics, Plainview, NY) for 15 min, at 4°C under a continuous nitrogen flow. Titanium particles were removed by centrifugation of the liposome suspension at 2,000 r/min for 5 min in a bench top centrifuge. The un-entrapped drug was separated from the liposome-entrapped drug by gel filtration on Sephadex G-50. Sterilization of the liposome suspension was accomplished by filtration through 0.4 and $0.2\,\mu m$ Nucleopore membranes (Nucleopore Corporation, Pleasanton, CA). The size range of the membrane-filtered vesicles as determined by transmission electron microscopy with phototungstic acid negative staining was 40 to 180 nm. The ADM content of liposomes was determined from samples diluted in acidified ethanol (0.3 N HCl in 50% ethanol) either fluorometrically with a Perkin-Elmer MPF 44 spectrofluorometer (excitation: 490 nm; emission: 590 nm) or by measuring the optical density with a Gilford spectrophotometer, at 496 nm wavelength. Fluorescence intensity and optical density were translated to $\mu g$ (fluorescence and absorbance) or ng (fluorescence only) of ADM-equivalents using standard curves of ADM. The characteristics of the liposome preparations have been previously discussed (Gabizon *et al.*, 1982). Briefly, 50–65% of the initial amount of ADM was retained in the liposomes using a 3:7 molar ratio of either phosphatidylserine (PS):phosphatidylcholine (PC) or phosphatidylglycerol (PG):PC, respectively. The final molar ratio of ADM to phospholipids in the vesicles was ~12%. Liposomes were stored at 4°C in sterile, siliconized, vacuum-sealed tubes (Vacutainer Systems, Rutherford, NJ) protected from light. Liposomes were used within 3 weeks after storage. Mice given injections of free ADM received freshly prepared drug solutions.

### *Histological examinations*

Light microscopy examinations were performed in organs from tumour-inoculated mice with and without treatment. Liver, spleen, and kidneys were fixed in Bouin's solution and stained with haematoxylin–eosin–phosphomolybdic acid: light green stain. For details of the staining procedure see Levi-Schaffer *et al.* (1982).

### *Statistical analysis*

The statistical significance of the results was evaluated by the non-parametric, ranking test of Wilcoxon.

### Results

### *Tumourigenic capacity of i.v. inoculated J-6456 cells*

Figure 1 shows linear regression analysis of the results obtained when the survival of mice challenged i.v. with growing inocula of tumour cells is plotted against the logarithm of the inoculum. One hundred tumour cells were sufficient to grow and eventually kill most of the animals (80–100% depending on the experiment). No tumour takes were observed after injection of 10 tumour cells. According to this experiment the estimated period of time required for one log increase of the J-6456 lymphoma is between 4 to 5 days.

**Figure 1** Linear regression of the size of tumour cell inoculum plotted against the mean survival time of inoculated mice. J-6456 cells were injected i.v. into BALB/c ♂ mice. No tumour takes were obtained in mice inoculated with $10^1$ tumour cells and observed for 60 days.

**Figure 2** Lack of enhancement of the anti-tumour effect by the simultaneous administration of free ADM and plain liposomes. Schedule of treatment indicated by arrows. The lipid dose of mice receiving plain liposomes and mice receiving L-ADM was the same: 6 μmol phospholipids and 6 μmol Chol per mouse. (●——●) Untreated mice; (○——○) Free ADM, 8 mg kg$^{-1}$; (○– – –○) Plain liposomes (PS:PC:Chol) and free ADM, 8 mg kg$^{-1}$; (○—··—○) L-ADM (PS:PC:Chol), 8 mg kg$^{-1}$.

**Figure 3** The effect of changes in the liposome composition on the antitumour activity of L-ADM. Schedule of treatment indicated by arrows. (●——●) Untreated mice; (○——○) Free ADM, 8 mg kg$^{-1}$; (●– – –●) L-ADM (PS:PC:Chol; molar ratio, 3:7:10), 8 mg kg$^{-1}$; (○– – –○) L-ADM (PS:PC:Chol; molar ratio, 3:7:2.5), 8 mg kg$^{-1}$; (●—·—·—●) L-ADM (PG:PC:Chol, molar ratio, 3:7:10), 8 mg kg$^{-1}$; (○— — —○) L-ADM (PG:PC:Chol; molar ratio, 3:7:2.5), 8 mg kg$^{-1}$.

**Figure 4** Superior therapeutic activity of L-ADM as compared to the maximal tolerated doses of free ADM using a single i.v. injection 3 days after tumour inoculation. (●——●) Untreated mice; (●– – –●) Free ADM, 12 mg kg$^{-1}$ (toxic dose); (○– – –○) Free ADM, 10 mg kg$^{-1}$ (subtoxic dose); (●—·—·—●) L-ADM (PG:PC:Chol), 12 mg kg$^{-1}$.

### *Comparison of the effects of L-ADM and free ADM treatments on the survival of tumour-inoculated mice*

The $10^6$ cell inoculum was chosen for the chemotherapeutic studies so that treatment efficacy could be tested on a reasonably high tumour burden. Chemotherapy was administered not less than 3 days after tumour injection to try not to interfere with tumour cell arrest in target tissues and initiation of proliferation. Table I shows the results of the treatment with free ADM and L-ADM at equal doses on the survival of mice inoculated i.v. with the J-6456 lymphoma. In the experiments presented in Table I, treatment consisted of at least 3 weekly injections using a non-toxic dose of ADM (8 mg kg$^{-1}$). The phospholipid dose per injection was 4–6 μmol per mouse. The administration of L-ADM resulted in a reproducible and significant prolongation of survival when compared to the effect of free ADM. Free ADM was also active against the J-6456

**Table I** Increased anti-tumour activity of L-ADM[a]

| | | | Survival time (days) | | |
|---|---|---|---|---|---|
| *Exp. no.* | | *No. of mice* | *Mean ± s.e.* | *Median (range)* | $P^b$ |
| 1 | Untreated | 5 | 21.0 ± 0.6 | 20.0 (20–22) | |
| | Free ADM | 7 | 29.0 ± 1.2 | 28.5 (24–32) | < 0.01 |
| | L-ADM (PS:PC:Chol) | 7 | 41.3 ± 1.7 | 41.0 (34–48) | < 0.01 |
| 2 | Untreated | 7 | 19.3 ± 0.5 | 18.5 (18–21) | |
| | Free ADM | 8 | 40.5 ± 2.3 | 40.0 (28–50) | < 0.01 |
| | L-ADM (PG:PC:Chol) | 8 | 71.1 ± 12.9 | 53.0 (41–148) | < 0.01 |
| 3 | Untreated | 8 | 19.5 ± 0.3 | 19.0 (18–21) | |
| | Free ADM | 9 | 32.1 ± 1.1 | 30.0 (30–40) | < 0.01 |
| | L-ADM (PG:PC:Chol) | 9 | 44.8 ± 1.3 | 43.5 (39–51) | < 0.01 |
| 4 | Untreated | 10 | 18.6 ± 0.4 | 18.1 (17–21) | |
| | Free ADM | 10 | 27.3 ± 0.6 | 26.5 (24–30) | < 0.01 |
| | L-ADM (PS:PC:Chol) | 8 | 48.5 ± 6.8 | 45.0 (22–77) | < 0.1 |
| 5 | Untreated | 10 | 23.3 ± 0.8 | 22.0 (24–28) | |
| | Free ADM | 15 | 38.6 ± 2.2 | 35.2 (31–60) | < 0.01 |
| | L-ADM (PS:PC:Chol) | 12 | 49.2 ± 2.9 | 48.0 (36–70) | < 0.01 |
| 6 | Untreated | 7 | 21.7 ± 1.8 | 19.8 (18–32) | |
| | Free ADM | 14 | 25.3 ± 0.6 | 23.9 (23–33) | < 0.01 |
| | L-ADM (PS:PC:Chol) | 15 | 32.4 ± 0.9 | 32.4 (26–41) | < 0.01 |

[a]BALB/c mice inoculated with $10^6$ J-6456 cells and treated i.v. with 8 mg kg$^{-1}$ ADM in either free or liposome-entrapped form according to the following schedule:

| | Days |
|---|---|
| Exp. 1: | 3, 10, 17 |
| Exp. 2: | 3, 10, 17 |
| Exp. 3: | 3, 10, 17 |
| Exp. 4: | 3, 10, 17, 24 |
| Exp. 5: | 3, 10, 17, 36, 46 |
| Exp. 6: | 11, 18, 25, 32. |

[b]Wilcoxon test: Free ADM analyzed *versus* Untreated; L-ADM analyzed *versus* Free ADM.

tumour but the increase in median life span observed in experiments 1 to 5 was in the range of 42–116% as compared to 105–187% for L-ADM treated mice. As expected the anti-tumour effect observed was more marked if treatment was started 3 days after tumour inoculation (Experiments 1 to 5) instead of 11 days after tumour inoculation (Experiment 6). Nevertheless, in the latter experiment L-ADM was still significantly superior to free ADM, indicating that the relative anti-tumour efficacy of the liposome-associated drug is not affected by the presence of a higher tumour burden. Another point worth noting in Table I is that increasing the number of treatments from 3 to 4 and 5 injections did not further improve the anti-tumour effects observed. A rough evaluation of the relative efficacies of these two modalities of treatment can be inferred from our data on the growth kinetics of the J-6456 lymphoma shown in Figure 1. Since the log growth time of this tumour is ~5 days, and the differences in mean and median survivals between free ADM-treated and L-ADM-treated mice of experiments 1 to 5 were >10 days, the estimated cytoreductive effect of L-ADM in this tumour model is ~100 times higher than that of free ADM. Exceptionally there were long-term survivors with no macroscopic tumour recognizable at autopsy among L-ADM treated mice only.

Association of ADM with liposomes was required for the enhancement of the anti-tumour effect to occur. As shown in Figure 2, free ADM injected together with plain liposomes (mixed immediately before injection) was not more effective than free ADM alone. This indicates that simultaneous administration of the drug with plain liposomes does not lead to a synergistic anti-tumour effect.

We also investigated the effects of reducing the Chol content of the liposomes and of substituting

PS by PG as the negatively-charged lipid component. The results of this experiment are presented in Figure 3. Decreasing the Chol:phospholipid molar ratio from 100% to 25% did not cause any change in the *in vivo* anti-tumour activity of the ADM-loaded liposomes. The survival curves shown in Figure 3 also indicate that there was no significant difference in the anti-tumour activity using either PS or PG liposomes. This finding is in agreement with the results obtained in Table I in separate experiments.

In Figure 4, we compare the anti-tumour effect of maximally tolerated doses of free ADM in single bolus injection to an equivalent dose of L-ADM. Using a dose of $12\,mg\,kg^{-1}$ of free ADM, a certain degree of toxicity is seen ($\sim$LD 10). With the same dose of L-ADM, no toxicity was observed and the anti-tumour effect was superior to the toxic dose ($12\,mg\,kg^{-1}$) and to the maximally tolerated dose ($10\,mg\,kg^{-1}$) of free ADM.

The therapeutic effects of L-ADM and free ADM were compared also on locally growing tumours obtained by i.m. inoculation of the J-6456 lymphoma. Mice received $10^6$ tumour cells i.m. into the thigh and were treated i.v. with $8\,mg\,kg^{-1}$ of either free ADM or L-ADM on days 3, 10 and 17 after tumour inoculation. Median survivals were 22.0 (s.d. = 3.3), 28.6 (s.d. = 1.5), and 36.0 (s.d. = 7.9) days respectively for untreated, L-ADM treated and free ADM treated mice. The differences between untreated and L-ADM treated, on the one hand, and between L-ADM treated and free ADM treated, on the other hand, were both statistically significant at the $P < 0.01$ level (Wilcoxon test). Thus clearly, free ADM was more effective than an equal dose of L-ADM against tumour cells growing locally in the i.m. site, while the superior antitumour effect of L-ADM was expressed on tumours located in selected anatomic areas such as liver and spleen.

*Histopathological study*

An anti-tumour experiment was conducted for the purpose of histopathological observations on tumour growth and response to treatment. Mice were inoculated i.v. with $10^6$ J-6456 tumour cells and 10 days later treated with either free or liposome-entrapped ADM given in a single i.v. shot at dose of $10\,mg\,kg^{-1}$. Groups of mice were sacrificed at days 10, 13, 17, 22 and 26 after tumour inoculation, for examination.

Untreated mice showed after treatment a progressive increase of liver and spleen weights from day 10 after tumour cell injection and of kidney weight from day 15 after tumour cell injection. The weights of the livers of dying mice were between 3.5 and 3.9 g as compared to the normal liver weight which did not exceed 1.4 g. The spleen and kidney weights reached respectively 400 mg (normal, 110 mg) and 650 mg (normal, 400 mg). Grossly, there was hepatosplenomegaly with focal areas of tumour involvement noticeable in the liver and sometimes also in the kidneys.

All treated mice showed extensive regression of tumour foci in the liver and spleen. Although the pattern and timing of tumour regression was similar with both treatment modalities, there was a noticeable difference in the extent and duration of the responses observed. As seen in Table II, complete pathologic tumour regression was found in only 2/12 mice treated with free ADM, whereas among L-ADM-treated mice examined in the same period of time, complete pathological remission was present in 9/12 animals. The data of Table II also suggest that the liposome associated drug was effective in preventing or delaying tumour development in the kidneys. Figures 5, 6 and 7 show representative microscopic cross-sections of the livers of untreated, free-ADM-treated and L-ADM-treated mice, 3 days after drug injection.

**Table II** Anti-tumour effects of L-ADM and free ADM on the J-6456 lymphoma: histopathological examinations.[a]

| Days after tumour inoculation | *No. of mice with microscopic tumour/no. of mice examined* | | | | | | | | |
|---|---|---|---|---|---|---|---|---|---|
| | *Untreated* | | | *Free ADM* | | | *L-ADM* | | |
| | *Liver* | *Spleen* | *Kidney* | *Liver* | *Spleen* | *Kidney* | *Liver* | *Spleen* | *Kidney* |
| 10 | 3/3 | 3/3 | 0/3 | — | — | — | — | — | — |
| 13 | 3/3 | 3/3 | 3/3 | 2/3 | 2/3 | 1/3 | 0/3 | 0/3 | 0/3 |
| 17 | 3/3 | 3/3 | 3/3 | 2/3 | 0/3 | 0/3 | 0/3 | 0/3 | 0/3 |
| 22 | | All dead | | 3/3 | 2/3 | 2/3 | 1/3 | 0/3 | 0/3 |
| 26 | | | | 3/3[b] | 3/3 | 3/3 | 2/3 | 1/3 | 2/3 |

[a]Treatment was administered 10 days after inoculation of the tumour and consisted of one i.v. injection of ADM, $10\,mg\,kg^{-1}$ in either free of liposome-encapsulated form.

[b]Two out of 3 mice found dead.

This study did not reveal any significant toxic effects on the normal liver and kidney tissues with either form of treatment. In the spleen, severe inhibition of erythro and myelopoiesis was observed with both types of treatment. Between days 7 and 12 after drug administration haemopoietic function of the spleen was restored to normal.

### Discussion

The present study indicates that a significantly increased life prolongation can be obtained using a liposome-associated form of ADM to treat a metastatic tumour with predominant spread to the liver and spleen. Increased antitumour activity of L-

**Figure 5** Representative field of a BALB/c mouse liver, 13 days after inoculation of $10^6$ J-6456 tumour cells. Multiple tumour cell foci with radial growth from the perivascular centrilobular area into the surrounding parenchyma are seen ( $\times$ 200).

**Figure 6** Representative field of a BALB/c mouse liver 13 days after inoculation of $10^6$ J-6456 lymphoma cells and 3 days after treatment with free ADM (10 mg $kg^{-1}$). A partially necrotic tumour cell focus is shown ( $\times$ 200).

**Figure 7** Representative field of a BALB/c mouse liver 13 days after inoculation of $10^6$ J-6456 lymphoma cells and 3 days after treatment with L-ADM ($10 mg kg^{-1}$). No tumour cells are found ($\times 200$).

ADM on intrahepatic metastases albeit with a phagocytic tumour, has been also shown by Mayhew *et al.* (1983). These results are in agreement with our previous data indicating increased intracellular drug levels in liver-residing tumour cells of mice treated with L-ADM as compared to free ADM (Gabizon *et al.*, 1983). The therapeutic advantage of L-ADM over free ADM rests apparently on a drug concentration effect in the involved tissues, although it remains questionable whether the drug is taken up by tumour cells directly in its liposome-associated form or indirectly after release from liposomes stored by the RES. In this context, the possibility of ADM transfer from macrophages into tumour cells has been reported (Martin *et al.*, 1982).

The activity of L-ADM appears to be dependent on tumour location in specific anatomic areas accessible to liposomes, such as liver and spleen. This point is emphasized by the reduced effectiveness of L-ADM found in mice with i.m. implanted tumours. The question as to whether anatomical barriers will determine differential access and differential anti-tumour activities of liposome delivered drugs in different body locations has raised objections about the applicability of these carriers in cancer chemotherapy (Poste, 1983). However, the therapeutic activity of L-ADM on tumour cells infiltrating the liver, spleen and, probably the kidneys, as shown here, is a distinct advantage which may outweight in selected neoplastic conditions a possible reduced activity in other locations.

It has been proposed that liposomes act by providing a slow-release drug depot, improving the pharmacokinetic properties of some drugs (Kaye *et al.*, 1981; Richardson & Ryman, 1982; Mayhew & Papahadjopoulos, 1983). With phase-specific drugs, such as cytosine arabinoside (Ara-C) and methotrexate, the higher and sustained blood levels appear to account for a superior antitumour activity when compared to free drug bolus injections (Richardson & Ryman, 1982). Indeed, it has been reported that there is no difference in the therapeutic efficacy comparing a single dose of liposomal Ara-C with a 5-day infusion of free Ara-C in an i.v.–tumour–i.v. treament model (Mayhew *et al.*, 1982). With regard to ADM, the factors regulating the therapeutic index of the liposome associated form seem to be different since it has been recently reported that the therapeutic index of 24 h infused free ADM is still inferior to that of L-ADM (Mayhew & Rustum, 1984). We have found previously (Gabizon *et al.*, 1982) that the blood levels of ADM are 10–100 times lower than those already detected in the liver and spleen 1 h after injection of ADM entrapped in negatively-charged liposomes. Therefore, it is unlikely that the circulating liposomal drug pool can account for a prolonged exposure of accessible tumour cells to ADM. However, a slow-release effect within the tumour-involved liver may well be important in determining the antitumour effect. In this context, we have shown that the hepatic clearance of ADM is prolonged in mice injected with the liposomal drug (Gabizon *et al.*, 1982). A similar finding was reported by Kaye *et al.* (1981) for Actinomycin-D.

ADM has been incorporated to liposomes of various compositions and prepared by different methods. Obviously standardization of the liposome composition and procedure of preparation is required if any pharmaceutical development is considered. The addition of negatively-charged phospholipids to PC ensures a high degree of drug capture by the liposome bilayer (Goormaghtigh *et al.*, 1980; Gabizon *et al.*, 1982). Based on these consideration, we chose to carry out the *in vivo* anti-tumour experiments with liposomes containing either PG or PS as negatively charged phospholipid. As shown in the present study, neither the replacement of PS with PG, nor the reduction of Chol content, significantly affected the anti-tumour effect observed. However, if therapeutic applications of L-ADM are contemplated, PG should be preferred to PS because it is less sensitive to lipid oxidation processes affecting the head group (Roseman *et al.*, 1975) and can be readily obtained from animal sources by a simpler enzymatic conversion of PC (Comfurius & Zwaal, 1977). The fact that the Chol:phospholipid molar ratio can be decreased to 25% without any significant loss of anti-tumour activity is consistent with the ability of liposomes to withstand the deleterious effect of serum proteins when the Chol content is kept above 20% (Pownall *et al.*, 1979; Snyder & Freire, 1980). This reduction of Chol content is important as it allows to reduce the lipid load necessary to deliver therapeutic doses of ADM.

In our studies, we have used sonication as a means of obtaining a small sized vesicle population with a reproducible size distribution. Small liposomes (<100 nm) can apparently reach the parenchymal area of the liver to a significant scale (Poste *et al.*, 1982) and are more efficiently incorporated by non-phagocytic cells when compared to large-sized vesicles (Straubinger *et al.*, 1983; Machy & Leserman, 1983; Matthay *et al.*, 1984), suggesting that they constitute the most advantageous vesicles for *in vivo* tissue penetrability and delivery of cytotoxic drugs to tumour cells. It is at present unclear whether drug-loaded vesicles of similar size prepared by different methods such as sonication, membrane extrusion, and French press (reviewed by Szoka & Papahadjopoulos, 1980) have the same biological activity.

Obviously, the potential clinical benefit of L-ADM must be evaluated in the light of the reduced acute and chronic toxicities reported with this modality of administration (Rahman *et al.*, 1980, 1982; Forssen & Tokes, 1981; Olson *et al.*, 1982; Herman *et al.*, 1983). Since the superior antitumour activity of L-ADM in the metastatic model is already apparent at equal doses of free drug, and the LD50 of ADM is significantly increased by liposome association (Olson *et al.*, 1982; Gabizon *et al.*, 1984), it can be inferred that liposome entrapment improves the therapeutic index of ADM in a selected group of neoplastic conditions. Yet it is not possible to predict at this time, the actual scope of clinical applicability in view of the broad heterogeneity of tumours with regard to factors such as microvascular architecture (Shubik, 1982), nodular *versus* diffuse growth, zonal differences in various phenotypic characteristics (Fidler & Hart, 1981), endocytic uptake of particular material and macrophage infiltration (Talmadge *et al.*, 1981), all of which may have important implications on accessibility and uptake of liposomes.

This work was supported by the Israel National Council for Research and Development.

### References

COMFURIUS, P. & ZWAAL, R.F.A. (1977). The enzymatic synthesis of phosphatidylserine and purification by CM-cellulose column chromatography. *Biochim. Biophys. Acta*, **488**, 36.

FIDLER, I.J. & HART, I.R. (1981). Biological and experimental consequences of the zonal composition of solid tumours. *Cancer Res.*, **41**, 3266.

FORSSEN, E.A. & TOKES, Z.A. (1979). *In vitro* and *in vivo* studies with Adriamycin liposomes. *Biochem. Biophys. Res. Commun.*, **91**, 1295.

FORSSEN, E.A. & TOKES, Z.A. (1981). Use of anionic liposomes for the reduction of chronic doxorubicin-induced cardiotoxicity. *Proc. Natl Acad. Sci.*, **78**, 1873.

FORSSEN, E.A. & TOKES, Z.A. (1983). Improved therapeutic benefits of doxorubicin by entrapment in anionic liposomes. *Cancer Res.*, **43**, 546.

GABIZON, A., DAGAN, A., GOREN, D., BARENHOLZ, Y. & FUKS, Z. (1982). Liposomes as *in vivo* carriers of Adriamycin: Reduced cardiac uptake and preserved antitumour activity in mice. *Cancer Res.*, **42**, 4734.

GABIZON, A., GOREN, D., FUKS, Z., BARENHOLZ, Y., DAGAN, A. & MESHORER, A. (1983). Enhancement of Adriamycin delivery to liver metastatic cells with increased tumoricidal effect using liposomes as drug carriers. *Cancer Res.*, **43**, 4730.

GABIZON, A. & TRAININ, N. (1980). Enhancement of growth of a radiation-induced lymphoma by T-cells from normal mice. *Br. J. Cancer*, **42**, 551.

GABIZON, A., MESHORER, A., FUKS, Z. & BARENHOLZ, Y. (1984). Enhanced antitumor activity and reduced toxicity of liposome-encapsulated Adriamycin. *Proc. Am. Assoc. Cancer Res.*, **25**, 302.

GOORMAGHTIGH, E., CHATELAIN, P., CASPERS, J. & RUYSSCHAERT, J.M. (1980). Evidence of a specific complex between Adriamycin and negatively-charged phospholipids. *Biochim. Biophys. Acta*, **597**, 1.

HERMAN, E.H., RAHMAN, A., FERRANS, V.J., VICK, J.A. & SCHEIN, P.S. (1983). Prevention of chronic doxorubicin cardiotoxicity in beagles by liposomal encapsulation. *Cancer Res.*, **43**, 5427.

KAYE, S.B., BODEN, J.A. & RYMAN, B.E. (1981). The effect of liposome entrapment of Actinomycin D and Methotrexate on the *in vivo* treatment of sensitive and resistant solid murine tumors. *Eur. J. Cancer*, **17**, 279.

LEVI-SCHAFFER, F., BERNSTEIN, A., MESHORER, A. & ARNON, R. (1982). Reduced toxicity of daunorubicin by conjugation to dextran. *Cancer Treat. Rep.*, **66**, 107.

MACHY, P. & LESERMAN, L.D. (1983). Small liposomes are better than large liposomes for specific drug delivery *in vitro*. *Biochim. Biophys. Acta*, **730**, 313.

MARTIN, F., CAIGNARD, A., OLSSON, O., JEANNIN, J.F. & LECLERC, A. (1982). Tumoricidal effect of macrophages exposed to Adriamycin *in vivo* or *in vitro*. *Cancer Res.*, **42**, 3851.

MATTHAY, K.K., HEATH, T.D. & PAPAHADJOPOULOS, D. (1984). Specific enhancement of drug delivery to AKR lymphoma by antibody-targeted small unilamellar vesicles. *Cancer Res.*, **44**, 1880.

MAYHEW, E., RUSTUM, Y. & VAIL, W.J. (1983). Inhibition of liver metastases of M5076 tumor by liposome-entrapped Adriamycin. *Cancer Drug Deliv.*, **1**, 43.

MAYHEW, E. & PAPAHADJOPOULOS, D. (1983). Therapeutic applications of liposomes. In *Liposomes*, p. 289. (Ed. Ostro) Marcel Dekker: New York & Basel.

MAYHEW, E., RUSTUM, Y.M. & SZOKA, F. (1982). Thereapeutic efficacy of cytosine arabinoside trapped in liposomes. In *Targeting of Drugs*, p. 249. (Eds. Gregoriadis *et al.*) Plenum Press: New York & London.

MAYHEW, E. & RUSTUM, Y. (1984). Toxicity and therapeutic efficacy of Adriamycin administered as an intravenous infusion or entrapped in liposomes in mice. *Proc. Am. Assoc. Cancer Res.*, **25**, 267.

OLSON, F., MAYHEW, E., MASLOW, D., RUSTUM, Y. & SZOKA, F. (1982). Characterization, toxicity and therapeutic efficacy of Adriamycin encapsulated in liposomes. *Eur. J. Cancer Clin. Oncol.*, **18**, 167.

PATTENGALE, P.K. & FRITH, C.H. (1983). Immunomorphologic classification of spontaneous lymphoid cell neoplasms occuring in female BALB/c mice. *J. Natl. Cancer Inst.*, **70**, 169.

POSTE, G. (1983). Liposome targeting *in vivo*: problems and opportunities. *Biol. Cell*, **47**, 19.

POSTE, G., BUCANA, C., RAZ, A., BUGELSKI, P., KIRSH, R. & FIDLER, I.J. (1982). Analysis of the fate of systemically administered liposomes and implications for their use in drug delivery. *Cancer Res.*, **42**, 1412.

POWNALL, H.J., MASSEY, J.B., KUSSEROW, S.K. & GOTTO, A.M. (1979). Kinetics of lipid-protein interactions: effect of cholesterol on the association of human plasma high-density apolipoprotein A–I with L-*h*-dimiristoyl-phosphatidyl-choline. *Biochemistry*, **18**, 574.

RAHMAN, A., KESSLER, A., MORE, N., SIKIC, B., ROWDEN, G., WOOLLEY, P. & SCHEIN, P.S. (1980). Liposomal protection of Adriamycin-induced cardiotoxicity in mice. *Cancer Res.*, **40**, 1532.

RAHMAN, A., MORE, N. & SCHEIN, P.S. (1982). Doxorubicin-induced chronic cardiotoxicity and its protection by liposomal administration. *Cancer Res.*, **42**, 1817.

RICHARDSON, V.J. & RYMAN, B.E. (1982). Effect of liposomally trapped antitumor drugs on a drug-resistant mouse lymphoma *in vivo*. *Br. J. Cancer*, **45**, 552.

ROSEMAN, M., LITMAN, B.J. & THOMPSON, T.E. (1975). Transbilayer exchange of phosphatidylethanolamine for phosphatidylcholine and N-acetimidoyl-phosphatidyl-ethanolamine in single-walled bilayer vesicles. *Biochemistry*, **14**, 4826.

SEGAL, A.W., WILLS, E.J., RICHMOND, J.E., SLAVIN, G., BLACK, C.D.V. & GREGORIADIS, G. (1974). Morphological observations on the cellular and subcellular destination of intravenously administered liposomes. *Br. J. Exp. Pathol.* **55**, 320.

SHUBIK, P. (1982). Vascularization of tumors: a review. *J. Cancer Res. Clin. Oncol.*, **103**, 211.

SNYDER, B. & FREIRE, E. (1980). Compositional domain structure in phosphatidyl-choline-cholestrol and sphingomyelin-cholesterol bilayers. *Proc. Natl. Acad. Sci.*, **77**, 4055.

STRAUBINGER, R.M., HONG, K., FRIEND, D.S. & PAPAHADJOPOULOS, D. (1983). Endocytosis of liposomes and intracellular fate of encapsulated molecules: encounter with a low pH compartment after internalization in coated vesicles. *Cell*, **32**, 1069.

SZOKA, F. & PAPAHADJOPOULOS, D. (1980). Comparative properties and methods of preparation of lipid vesicles (liposomes). *Annu. Rev. Biophys. Bioeng.*, **9**, 467.

TALMADGE, J.E., KEY, M. & FIDLER, I.J. (1981). Macrophage content of metastatic and nonmetastatic rodent neoplasms. *J. Immunol.*, **126**, 2245.